# Daily combined measurement of platelet count and presepsin concentration can predict in-hospital death of patients with severe coronavirus disease 2019 (COVID-19)

**DOI:** 10.1007/s12185-023-03555-5

**Published:** 2023-03-15

**Authors:** Hiroyasu Ishikura, Junichi Maruyama, Maiko Nakashio, Kota Hoshino, Shinichi Morimoto, Yoshito Izutani, Junta Noake, Takahiro Yamagaito, Maho Yoshida, Taisuke Kitamura, Yoshihiko Nakamura

**Affiliations:** 1 Department of Emergency and Critical Care Medicine, Faculty of Medicine, Fukuoka University, 7-45-1 Nanakuma, Jonan-ku, Fukuoka, 814-0180 Japan; 2 Sysmex Product Marketing, 1-2-2 Osaki, Shinagawa-ku, Tokyo, Japan; 3 Sysmex Scientific Affairs, 1-3-2 Murotani, Nishi-ku, Kobe, Japan

**Keywords:** SARS-CoV-2, COVID-19, Coagulopathy, Platelet count, Presepsin, Prognosis

## Abstract

The purpose of this study was to classify patients with severe COVID-19 into more detailed risk groups using coagulation/fibrinolysis, inflammation/immune response, and alveolar/myocardial damage biomarkers, as well as to identify prognostic markers for these patients. These biomarkers were measured every day for eight intensive care unit days in 54 adult patients with severe COVID-19. The patients were classified into survivor (*n* = 40) and non-survivor (*n* = 14) groups. Univariate and multivariate analyses showed that the combined measurement of platelet count and presepsin concentrations may be the most valuable for predicting in-hospital death, and receiver operating characteristic curve analysis further confirmed this result (area under the curve = 0.832). Patients were consequently classified into three groups (high-, medium-, and low-risk) on the basis of their cutoff values (platelet count 53 × 10^3^/µL, presepsin 714 pg/mL). The Kaplan–Meier curve for 90-day survival by each group showed that the 90-day mortality rate significantly increased as risk level increased (*P* < 0.01 by the log-rank test). Daily combined measurement of platelet count and presepsin concentration may be useful for predicting in-hospital death and classifying patients with severe COVID-19 into more detailed risk groups.

## Introduction

Substantial progress in clinical research has led to a better understanding of severe acute respiratory syndrome coronavirus (SARS-CoV-2) and the management of coronavirus disease 2019 (COVID-19). However, SARS-CoV-2 continues to wreak havoc worldwide, with many countries enduring repeated outbreaks of this viral illness, attributed mainly to the emergence of mutant variants of the virus [1].

To date, our Extracorporeal Membranous Oxygenation (ECMO) Center has managed more than 80 severe COVID-19 patients in over three years. During that time, we have observed that there are three general types of patients with severe COVID-19: one type recovers well with appropriate intensive care unit (ICU) treatment, another type recovers with some difficulty with extensive ICU treatment, and the last type of patient dies despite thorough treatment in the ICU. On the basis of these observations, we hypothesized that patients with severe COVID-19 may be classifiable into more finely detailed severity categories.

The measurement of sepsis biomarkers represents a cornerstone in the risk assessment of patients with severe infectious diseases and/or sepsis because these biomarkers can help predict clinical progression and guide therapeutic management [2]. Not surprisingly, patients with severe COVID-19 are frequently complicated by characteristic coagulation/fibrinolysis abnormalities [3–5]. These abnormalities are called COVID-19-associated coagulopathy (CAC). An elevated D-dimer concentration and decreased platelet count occur more frequently in critically ill patients and may be correlated with increased mortality [6, 7].

We hypothesized that evaluating the severity of disease via the use of risk stratification markers in patients with severe COVID-19 may provide an early understanding of prognosis and help identify patients who require more medical device support, which may help improve their prognosis.

The purpose of this study was to determine the characteristics of CAC using biomarkers of coagulation/fibrinolysis, inflammation/immune response, and alveolar/myocardial damage, and to identify prognostic markers in patients with serious COVID-19 from these biomarkers. Moreover, we measured these biomarkers daily to determine how they changed over time and attempted to identify biomarkers that can classify severe COVID-19 patients in more detail.

## Patients and methods

This retrospective, single-center, observational study was conducted at the ECMO Center, Fukuoka University Hospital, Fukuoka, Japan, which is a 915-bed referral and tertiary hospital, from April 2020 to May 2021. The following ethics review boards approved the protocol for this study: Fukuoka University Hospital (U20-10-008; registered on 22 October 2020) and Sysmex Corporation (2020-242; registered on 13 November 2020). We obtained signed informed consent from the patients or proxy in accordance with the Declaration of Helsinki for publication of this study. Patients with severe COVID-19 aged ≥ 18 years who were diagnosed with SARS-CoV-2 infection detected by reverse transcription-polymerase chain reaction from a nasopharyngeal swab sample were enrolled in the study. Severe COVID-19 was defined as the condition in which patients were admitted to the ICU for COVID-19-induced sepsis with acute respiratory distress syndrome (ARDS) and they required mechanical ventilation management or additional ECMO management. Patients were evaluated for the presence of sepsis according to the Sepsis-3 diagnosis criteria [8] and for the presence of ARDS according to the Berlin definition [9]. The illness severity was evaluated by the Acute Physiology and Chronic Health Evaluation (APACHE) II score [10]. Organ failure was assessed by the Sequential Organ Failure Assessment (SOFA) score [11]. These scores are useful for evaluating the morbidity and mortality of critical illnesses.

Patients with severe COVID-19 were divided into the survivor group and the non-survivor group. The survivor group was defined as the absence of in-hospital death and the non-survivor group as the presence of in-hospital death.

### Study procedures

In patients with severe COVID-19, blood samples were routinely collected for measuring markers, and there was no lack of data on ICU admission in this study. We collected peripheral blood and measured coagulation/fibrinolysis biomarkers, such as the platelet count, PT-international normalized ratio (PT-INR), activated partial thromboplastin time (APTT), fibrinogen (Fbg), fibrin/fibrinogen degradation products (FDP), D-dimer, thrombin–antithrombin complex (TAT), plasmin α2-plasmin inhibitor complex (PIC), antithrombin (AT) activity, protein C (PC), thrombomodulin (TM), soluble fibrin (SF), and plasminogen activator inhibitor-1 (PAI-1). We also measured inflammation/immune response markers, which comprised the white blood cell (WBC) count, C-reactive protein (CRP), presepsin (P-SEP), procalcitonin (PCT), interleukin (IL)-6, IL-10, IL-18, monokine induced by interferon-γ (MIG), and macrophage inflammatory protein (MIP)-1α. We also measured the Krebs von den Lungen (KL)-6 and surfactant protein A (SP-A) concentrations as markers of alveolar damage [12, 13], and the N-terminal pro B-type natriuretic peptide (NT-proBNP) and troponin T (TnT) concentrations as markers of myocardial damage [14, 15]. Additionally, we measured total bilirubin, creatinine, lactate dehydrogenase, and ferritin concentrations.

### Statistical analysis

Unless otherwise indicated, all data are expressed as the median (interquartile range). SPSS 15.0 J (SPSS Inc., Chicago, IL, USA) was used for statistical analyses. Nonparametric statistical tests were used because these tests are more appropriate than traditional parametric tests for analysis of data sets with a high variability. Differences in biomarker levels between the two groups (survivors and non-survivors) were analyzed using the Mann–Whitney *U* test. Comparisons between three or more groups were carried out using the Bonferroni method.

The variables associated with in-hospital death by univariate analysis (*P* < 0.05) were introduced into a logistic regression model to perform a multivariable analysis with a Cox regression model analysis method. Variables with *P* < 0.1 in multivariate analysis were considered to be associated with in-hospital death. Receiver operated curve (ROC) analyses using the biomarkers with *P* < 0.05 in the univariate analysis were performed to assess their relationships with in-hospital death, and area under the curve (AUC) analysis was performed to assess the ability of these biomarkers to predict in-hospital death. We also calculated the cutoff values for associations with in-hospital death for these markers. Categorical data were compared using the Kaplan–Meier curves and were analyzed using log-rank tests to compare groups in terms of survival. Unless otherwise indicated, the level of statistical significance was set at *P* < 0.05.

## Results

### Population characteristics

Fifty-four patients with severe COVID-19 were enrolled and all patients were included in the analysis. The demographic and clinical characteristics of patients with severe COVID-19 are shown in Table 1. The median age of the patients (44 men; 10 women) was 60 (54–68) years. Among all patients, 40 were classified into the survivor group and 14 were classified into the non-survivor group (survival rate: 74.1%). The non-survivor group was significantly older than the survivor group (*P* < 0.01). Moreover, the non-survivor group had significantly higher APACHE II (*P* < 0.01) and SOFA (*P* = 0.02) scores than the survivor group. The non-survivor group also had significantly higher rates of continuous renal replacement therapy (*P* < 0.01) than the survivor group. Additionally, there was no difference between the two groups regarding past history, intervention except continuous renal replacement therapy, and contents of medication.

**Table 1:** Demographics and clinical characteristics of patients with severe COVID-19

Variable		Total patients (*n* = 54)	Survivor group (*n* = 40)	Non-survivor group (*n* = 14)	*P* value
Demographics	Age (years)	60 (54–68)	58 (52–63)	68 (64–72)	< 0.01
	Sex, male/female	44/10	32/8	12/2	0.65
	APACHE II score	12 (9–16)	11 (8–14)	16 (13–19)	< 0.01
	SOFA score	5 (4–7)	5 (4–6)	7 (4–11)	0.02
	PaO_2_/FiO_2_ at ICU admission	96 (72–133)	96 (70–129)	97 (73–154)	0.46
Past history	COPD (%)	13.0	12.5	14.3	0.88
	Chronic renal disease (%)	9.3	5.0	21.4	0.07
	DM (%)	42.6	42.5	42.9	0.99
	Cardiovascular disease (%)	18.5	17.5	21.4	0.76
	Post-organ transplantation or other immunodeficiency (%)	0.0	0.0	0.0	–
	Smoking history (%)	63.3	58.3	76.9	0.24
	Under treatment for malignancy (%)	1.9	0.0	7.1	0.10
Intervention	Prone position (%)	94.4	95.0	92.9	0.78
	Mechanical ventilation (%)	100.0	100.0	100.0	–
	ECMO (%)	57.4	50.0	78.6	0.07
	PMX-DHP (%)	24.1	17.5	42.9	0.06
	CRRT (%)	31.5	15.0	78.6	< 0.01
Medication	Favipiravir (%)	25.9	20.0	42.9	0.10
	Remdesivir (%)	81.5	87.5	64.3	0.06
	Dexamethasone (%)	85.2	90.0	71.4	0.10
	Heparin (%)	100.0	100.0	100.0	–

*COVID*-19 coronavirus disease 2019, *APACHE* acute physiology and chronic health evaluation, *SOFA* sequential organ failure assessment, *ICU* intensive care unit, *COPD* chronic obstructive pulmonary disease, *DM* diabetes mellitus, *ECMO* extracorporeal membrane oxygenation, *PMX-DHP* polymyxin B immobilized fiber column direct hemoperfusion, *CRRT* continuous renal replacement therapy

The Mann–Whitney U test was used for comparison between the survivor and non-survivor groups

### Distribution of various molecular biomarkers

Differences in baseline biomarker levels, such as coagulation/fibrinolysis, inflammation/immune response, and alveolar/myocardial damage markers, at ICU admission between the survivor and non-survivor group are shown in Table 2. With regard to coagulation/fibrinolysis markers, the non-survivor group had a significantly lower platelet count (*P* < 0.01) than the survivor group. Both the survivor group and the non-survivor group had normal or mildly deviated median PT**-**INR, APTT, PAI-1, and AT. With regard to inflammation/immune response markers, the non-survivor group had significantly higher P-SEP (*P* = 0.04) and IL-10 (*P* < 0.01) concentrations than the survivor group. With regard to alveolar/myocardial damage markers, the non-survivor group had significantly higher KL-6 (*P* = 0.03) concentrations than the survivor group.

**Table 2:** Various biomarkers markers in patients with severe COVID-19 for survivors and non-survivors

Category	Variable	Normal range or cut-off value	Survivor group (*n* = 40)	Non-survivor group (*n* = 14)	*P* value
Coagulation or fibrinolysis	PLT count (× 10^3^/µL)	15.3–34.8	227 (171–279)	147 (115–193)	< 0.01
	PT-INR	0.85–1.15	1.10 (1.07–1.17)	1.11 (1.00–1.22)	0.75
	APTT (s)	24.0–38.0	31.2 (27.8–35.8)	33.2 (28.6–51.1)	0.33
	Fbg (mg/dL)	200–400	620 (480–777)	574 (314–688)	0.24
	FDP (μg/mL)	< 5	8 (5–18)	16 (5–31)	0.38
	D-dimer (ng/µL)	< 1.0	2.8 (1.3–7.3)	6.2 (1.6–14.1)	0.21
	TAT (ng/mL)	< 3.0	4.8 (2.3–11.4)	7.3 (4.7–33.4)	0.10
	PIC (mg/dL)	< 0.8	2.0 (1.3–3.8)	2.3 (1.4–4.5)	0.80
	PAI-1 (mg/dL)	< 50	52 (32–78)	36 (29–109)	0.23
	AT (%)	80–130	92 (82–108)	90 (82–103)	0.66
	SF (μg/mL)	< 6.1	13.9 (8.5–32.7)	14.4 (8.3–104.7)	0.81
Inflammation or immune response	WBCs (/µL)	3.3–8.6	9.0 (6.2–12.7)	9.8 (7.1–17.4)	0.31
	CRP (mg/dL)	0.0–0.14	8.87 (3.04–15.28)	8.40 (4.60–22.25)	0.55
	P-SEP (pg/mL)	< 500	450 (353–687)	919 (374–3097)	0.04
	PCT (ng/mL)	< 0.3	0.16 (0.08–0.42)	0.21 (0.10–4.81)	0.27
	IL-6 (pg/mL)		67.84 (30.82–402.75)	393.63 (50.22–3304.55)	0.16
	IL-10 (pg/mL)		16.64 (10.43–40.88)	41.85 (30.36–71.54)	< 0.01
	IL-18 (pg/mL)		915.36 (729.53–1246.38)	798.41 (683.72–980.31)	0.33
	MIG (pg/mL)		121.11 (65.83–337.91)	219.33 (99.94–534.81)	0.35
	MIP-1α (pg/mL)		44.81 (27.13–87.17)	68.14 (30.45–148.40)	0.37
Alveolar damage	KL-6 (U/mL)	< 500	514 (307–902)	884 (502–2008)	0.03
	SP-A (ng/mL)	< 43.8	65.2 (41.3–100.0)	80.7 (42.1–111.1)	0.53
Myocardial damage	NT-proBNP (pg/mL)	< 125	326 (136–784)	1198 (198–3623)	0.16
	TnT (ng/mL)	< 0.016	0.016 (0.008–0.040)	0.023 (0.007–0.064)	0.66

The Mann**–**Whitney *U* test was used for comparison between the survivor and non-survivor groups

*COVID*-19 coronavirus disease 2019, *PLT* platelet, *PT-INR* prothrombin time**–**international normalized ratio, *APTT* activated partial thromboplastin time, *Fbg* fibrinogen, *FDP* fibrin/fibrinogen degradation products, *TAT* thrombin**–**antithrombin complex, *PIC* plasmin α2-plasmin inhibitor complex, *PAI-1* plasminogen activator inhibitor-1, *AT* antithrombin, *SF* soluble fibrin, *WBCs* white blood cells, *CRP* C-reactive protein, *P-SEP* presepsin, *PCT* procalcitonin, *IL* interleukin, *MIG* monokine induced by interferon-gamma, *MIP* macrophage inflammatory protein, *KL-6* Krebs von den Lungen-6, *SP-A* surfactant protein-A, *NT-proBNP* N-terminal pro B-type natriuretic peptide, *TnT* troponin T

### Association of biomarker levels with in-hospital death

We then performed univariate analysis to evaluate the predictive power for in-hospital death using the four above-mentioned biomarkers that were significant (*P* < 0.05) between the survivor and non-survivor groups on ICU admission. The platelet count, P-SEP, and KL-6 showed predictive power, with *P* < 0.05 as the cutoff for significance (Table 3). Additionally, we performed the multivariate analysis as covariates to determine the factors associated with in-hospital death using these three markers. P-SEP was the only factor associated with in-hospital mortality (*P* = 0.034).

**Table 3:** Univariate logistic regression model multivariable Cox regression model analysis for predictors of in-hospital mortality

Category	Variable	Univariate logistic regression model analysis				Multivariable cox regression model analysis		
		*P* value	Odds ratio	95% CI	ROC analysis AUC	*P* value	Hazard ratio	95% CI
Coagulation or fibrinolysis	PLT count (× 10^3^/µL)	< 0.001	0.9846	0.9739–0.9954	0.786	0.324	0.9951	0.9850–1.0049
Inflammation orImmune response	P-SEP (pg/mL)	0.005	1.0006	1.0000–1.0012	0.687	0.034	1.0004	1.0000–1.0007
	IL-10 (pg/mL)	0.507	1.0026	0.9951–1.0102	–	–	–	ー
Alveolar damage	KL-6 (U/mL)	0.019	1.0009	1.0001–1.0017	0.700	0.524	0.9998	0.9989–1.0005

*CI* confidence interval, *ROC* receiver operating characteristic curve, *AUC* area under the curve, *PLT* platelet, *P-SEP* presepsin, *IL* interleukin, *KL-6* Krebs von den Lungen-6

We then performed a ROC analysis using the platelet count, P-SEP, and KL-6, and calculated the AUC reported as a quantification of this analysis. The AUC of the platelet count was 0.786, that of P-SEP was 0.687, and that of KL-6 was 0.700 (Table 3). The value of AUC for all variables was below 0.8. From the values of the AUC, we concluded that a single biomarker alone did not provide sufficient power to predict in-hospital death. We then combined P-SEP, which was the only marker with predictive power for in-hospital mortality in multivariate analysis, with platelet count or KL-6, and performed a ROC analysis including the AUC for assessing in-hospital death.

This analysis selected the P-SEP concentrations and platelet count as the highest predictive markers for in-hospital death, with an AUC = 0.832 (sensitivity: 64.3%, specificity: 90.0%). The AUC of the other combination, P-SEP and KL-6, was 0.775.

The cutoff values of in-hospital death for these two markers identified by the ROC analysis were 153 × 10^3^/µL for the platelet count (sensitivity: 64.3%, specificity: 82.5%) and 714 pg/mL for P-SEP concentrations (sensitivity: 64.3%, specificity: 77.5%).

### Selection of a multi-marker panel and development of a COVID-19 severity classification algorithm

**Fig. 1:**
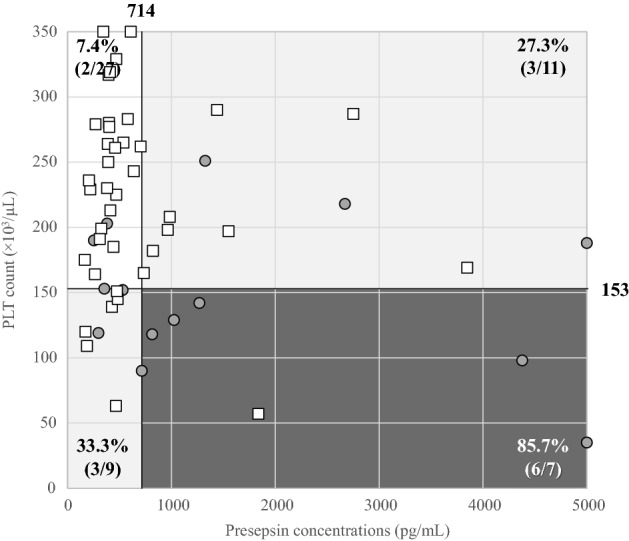
Severity and classification panel of patients with serious COVID-19 patients. Open squares indicate the survivor group, and closed circles indicate the non-survivor group. Patients were classified into the following four panels according to the cutoff value from platelet count and presepsin levels: (1) platelet count ≤ 153 × 10^3^/µL and P-SEP ≥ 714 pg/mL; (2) platelet count > 153 × 10^3^/µL and P-SEP ≥ 714 pg/mL; (3) platelet count ≤ 153 × 10^3^/µL and P-SEP < 714 pg/mL; and (4) platelet count > 153 × 10^3^/µL and P-SEP < 714 pg/mL. Categories of serious COVID-19 patients were classified as follows: **High-risk group**; dark gray area (1), **Medium-risk group**; gray area (2) + (3), and **Low-risk group**; white area (4). In-hospital mortality of the high-risk, medium-risk, and low-risk groups was 85.7% (6/7), 30.0% (6/20), and 7.4% (2/27), respectively. *COVID-19* coronavirus disease 2019, *PLT* platelet

In this study, the following biomarkers were the optimal biomarker panel for predicting in-hospital mortality: (1) platelet count (a coagulation marker) and (2) P-SEP (an inflammatory biomarker). The patients enrolled in this study were consequently divided into four panels according to these cutoff values (Fig. 1). The in-hospital mortality rate of each panel was as follows: (1) platelet count ≤ 153 × 10^3^/µL and P-SEP concentrations ≥ 714 pg/mL, 85.7% (6/7); (2) platelet count > 153 × 10^3^/µL and P-SEP concentrations ≥ 714 pg/mL, 27.3% (3/11); (3) platelet count ≤ 190 × 10^3^/µL and P-SEP concentrations < 714 pg/mL, 33.3% (3/9); and (4) platelet count > 190 × 10^3^/µL and P-SEP concentrations < 714 pg/mL, 7.4% (2/27). We then classified the patients into the following three categories according to the platelet count and P-SEP concentrations as follows: (1) high risk (*n* = 7), platelet count ≤ 153 × 10^3^/µL and P-SEP concentrations ≥ 714 pg/mL; (2) medium risk (*n* = 20), platelet count > 153 × 10^3^/µL and P-SEP concentrations ≥ 714 pg/mL, or platelet count ≤ 153 × 10^3^/µL and P-SEP concentrations < 714 pg/mL; and (3) low risk (*n* = 27), platelet count > 153 × 10^3^/µL and P-SEP concentrations < 714 pg/mL (Fig. 1).

The in-hospital mortality rate of patients in each group was 85.7% (6/7) in the high-risk group, 30.0% (6/20) in the medium-risk group, and 7.4% (2/27) in the low-risk group. The in-hospital mortality significantly increased (*P* < 0.01) as the risk of the group increased (Fig. 1).

### Illness severity according to the classification of in-hospital death

**Fig. 2:**
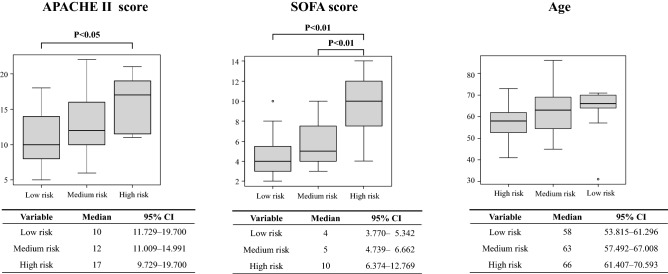
Comparison of APACHE II score, SOFA score, and age for each severity group classified by platelet count and presepsin levels. Patients were categorized into three groups according to the severity defined in the panel (Fig. 1); the APACHE II score and the SOFA score on ICU admission significantly increased as the COVID-19 severity increased. There was no significant difference in age between the three groups. However, the age increased as the risk of the group increased. The Bonferroni method was used to compare the three groups

**Fig. 3:**
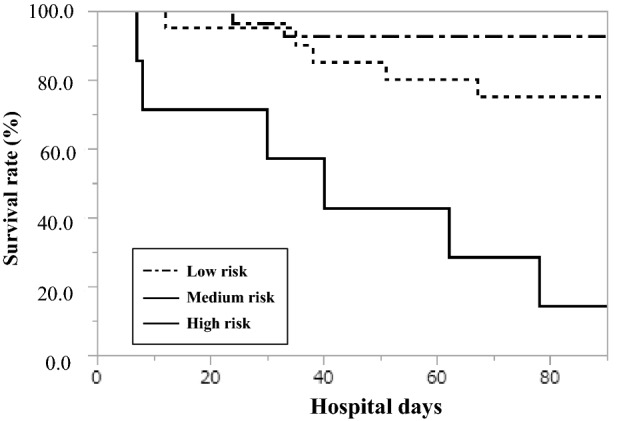
Kaplan–Meier survival plots for survival stratified by severity for the 90-day period. The survival rate was significantly higher in the low-risk group than in the moderate-risk and high-risk groups (*P* < 0.01 by log-rank test)

We propose the COVID-19 severity panel as a clinically useful numerical representation of the results from our multi-marker panel, which includes the platelet count and P-SEP panel testing. Additionally, to evaluate the diagnostic accuracy of this panel, we investigated whether this panel was able to stratify patients according to physiological severity, the degree of organ failure, age, and 90-day mortality. When patients were categorized into three groups according to the severity defined in this panel, the APACHE II score and the SOFA score on ICU admission significantly increased as the COVID-19 severity increased (Fig. 2). Age was not significantly different between the three groups. However, the risk of the group increased as age increased. The Kaplan–Meier curve for 90-day survival by each of the three groups derived from the panel is shown in Fig. 3. The survival rate was significantly higher in the low-risk group than in the medium-risk group and high-risk group (both *P* < 0.01 by the log-rank test).

### Time course of the distribution of various biomarkers

**Fig. 4:**
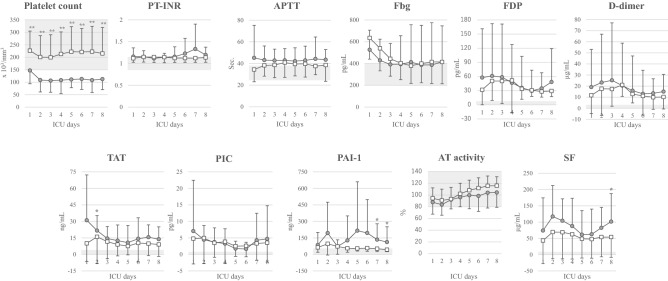
Daily changes in coagulation/fibrinolysis markers for each outcome. Open squares indicate the survivor group, and closed circles indicate the non-survivor group. Only platelet counts were significantly lower in the non-survivor group than in the survivor group on all days throughout the 8 days. *P < 0.05, **P < 0.01 for comparisons between the linked groups. The gray area indicates the normal range

**Fig. 5:**
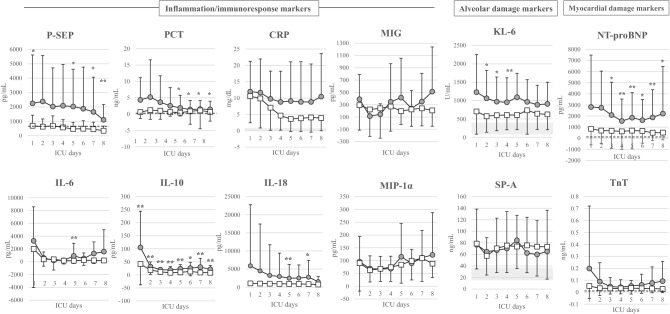
Daily changes in inflammation/immune response markers and alveolar/myocardial damage markers for each outcome. Open squares indicate the survivor group, and closed circles indicate the non-survivor group. In inflammation/immune response markers, only IL-10 was significantly higher in the non-survivor group than in the survivor group on all days throughout the 8 days. P-SEP was significantly higher in the non-survivor group than in the survivor group in 4 of the 8 days, excluding Days 2, 3, 4 and 6. PCT was also significantly higher in the non-survivor group than in the survivor group in 4 of the 8 days, excluding initial 4 days of Day 1 to Day 4. In alveolar/myocardial damage markers, NT-proBNP concentrations were significantly higher in the non-survivor group than in the survivor group in 6 of the 8 days, excluding Days 1 and 2. The gray area indicates the normal range. The dotted horizontal line indicates the cutoff level of each marker. We used 647 × 10^2^ pg/mL of P-SEP as a cutoff value in this study, which we previously reported as a cutoff value for diagnosing sepsis. *P < 0.05, **P < 0.01 for comparisons between the linked groups

The daily changes in the distribution of various molecular biomarkers up to Day 8 in the ICU are shown in Fig. 4 (11 coagulation/fibrinolytic markers) and Fig. 5 (eight inflammation/immune response markers, two alveolar damage markers, and two myocardial damage markers).

#### Coagulation/fibrinolysis markers

Daily changes in the value of coagulation/fibrinolysis markers for 8 days after ICU admission are shown in Fig. 4. Only the platelet count was significantly lower in the non-survivor group than in the survivor group on all 8 days. TAT, PAI-1, and SF concentrations were significantly different between the survivor and non-survivor groups only on 1 or 2 days throughout the 8 days. However, this result was not helpful in distinguishing both groups early in the ICU stay. There were no significant differences in the other markers (PT-INR, APTT, Fbg, FDP, D-dimer, PIC, and AT) between the two groups over the 8-day period. Based on the above, the initial values of coagulation/fibrinolysis markers other than platelet count during the ICU stay were not predictive of the patients’ outcomes. And for platelet count, the values in the survivor group remained within the normal range whereas those in the non-survivor group remained below the lower limit of the normal range throughout the 8 days.

#### Inflammation/immune response markers

Daily changes in the value of inflammation/immune response markers for 8 days after ICU admission are shown in Fig. 5. Only IL-10 concentrations were significantly higher in the non-survivor group than in the survivor group on all 8 days. P-SEP concentrations were significantly higher in the non-survivor group than in the survivor group on 4 of the 8 days, excluding Days 2, 3, 4, and 6. PCT concentrations were significantly higher in the non-survivor group than in the survivor group on 4 of the 8 days, excluding Days 1 to 4. IL-6 and IL-18 concentrations were significantly different between the survivor and non-survivor groups on only 1or 2 days throughout the 8 days. Other markers (CRP, MIG, and MIP-1α) were not significantly different between the two groups over the 8-day period. The initial values of all biomarkers excluding P-SEP and IL-10 were not significantly different between the survivor group and non-survivor group. Only for P-SEP concentration, the values in the survivor group remained below mostly the cutoff value, previously reported for sepsis diagnosis (600–800 pg/mL) [16–19], whereas those in the non-survivor group clearly remained above the cutoff value throughout the 8 days. At this time, there is no clear cutoff value for IL-10, an anti-inflammatory cytokine.

#### Alveolar/myocardial damage markers

Daily changes in the value of alveolar/myocardial damage markers for 8 days after ICU admission are shown in Fig. 5. KL-6 concentrations were significantly higher in the non-survivor group than in the survivor group on 3 of the 8 days. NT-proBNP concentrations were significantly higher in the non-survivor group than in the survivor group on 6 of the 8 days, excluding Days 1 and 2. However, initial values of both KL-6 and NT-proBNP were not significantly different between the survivor group and non-survivor group. Therefore, this result was not helpful in distinguishing these groups early during the ICU stay. SP-A and TnT concentrations were not significantly different between the two groups over the 8-day period.

## Discussion

The new infectious disease COVID-19 currently has no established curative treatment, and has a high mortality rate when it becomes severe. Therefore, the identification of biomarkers that can predict not only the severity but also the prognosis of this disease early on admission to the ICU is important for guiding the treatment direction and saving patients’ lives.

Patients with severe COVID-19 frequently experience coagulopathy as a complication. In this study, the non-survivor group had a significantly lower platelet count than the survivor group at admission to the ICU. However, the survivor and non-survivor groups had normal or mildly deviated median PT**-**INR, APTT, PAI-1, and AT. This result suggests that the pattern of COVID-19-associated coagulopathy is different from the pattern of conventional sepsis-induced coagulopathy, which usually presents with suppressed-fibrinolytic-type coagulopathy [20].

This study showed that a decrease in the platelet count, which is a coagulation marker, and an increase in concentrations of P-SEP, which is an inflammation marker, were valuable indicators for predicting a poor prognosis and the time course of a critical condition. In other words, we found that the prognosis of patients with severe COVID-19 was significantly worse if inflammation and coagulation were enhanced simultaneously. Additionally, measuring these two markers simultaneously and applying those levels to our proposed panel provided a high probability of diagnosing in-hospital death. To date, there have been very few reports limited to critically ill patients with COVID-19 [21]. Under severe conditions, patients in the high-risk group (with platelet count ≤ 153 × 10^3^/µL and P-SEP concentrations ≥ 714 pg/m) have an unexpectedly high in-hospital mortality rate of 85.7%. In contrast, low-risk groups (with platelet count > 153 × 10^3^/µL and P-SEP concentrations < 714 pg/mL), and even those patients with severe COVID-19 treated by mechanical ventilation or ECMO, have a low in-hospital mortality rate of 7.4%.

Thrombocytopenia is frequently the initial feature in sepsis [22]. Additionally, mortality of these patients is associated with prolonged thrombocytopenia and the absence of a relative increase in the platelet count [23].

We confirmed thrombocytopenia also occurs in patients with COVID-19 with severe disease and is potentially correlated with an increased mortality rate. In this study, a low platelet count on ICU admission (cutoff value: 153 × 10^3^/µL) that remained low without increasing throughout the first week of stay in the ICU had a poor prognosis. This finding suggests that daily measurement of the platelet count is a useful and essential tool for predicting in-hospital death. Additionally, this cutoff value is similar to the predicted value for bleeding complications reported by Al-Samkari et al. [24].

The measurement of P-SEP is reported to be useful for the initial diagnosis of sepsis, evaluating the severity of sepsis, risk stratification, and monitoring clinical responses to therapeutic interventions [16–19]. Recently, studies have reported that elevated concentrations of P-SEP may be a useful biomarker in the prognostic assessment of patients with COVID-19 [25, 26]. Additionally, in a population of patients with COVID-19 and acute respiratory failure in the emergency department, P-SEP was an accurate predictor of 30-day mortality [27, 28]. A previous study also showed that P-SEP values were increased by 2.74-fold in patients with COVID-19 with severe/critical illness compared with those without COVID-19 [29]. To date, there have been no reports of elevated P-SEP concentrations during viral infection. There have been a few reports that showed no increase in P-SEP concentrations under an influenza virus infection [30, 31]. However, these cases were not severe. In our study of all patients with severe COVID-19, survivors had significantly higher P-SEP concentrations than non-survivors throughout the first week of their stay in the ICU.

In this study, we found that patients with severe COVID-19 who had high P-SEP values on ICU admission (cutoff value: 714 pg/mL) that remained high without decreasing throughout the first week of their stay in the ICU had a poor prognosis. Previous reports showed a predicted cutoff value of 600–800 pg/mL for the diagnosis of sepsis [16–19], which was in close agreement with the cutoff value we found for in-hospital death in patients with severe COVID-19. This finding suggests that daily measurement of the P-SEP is also a useful and essential tool for predicting in-hospital death.

We want to describe the details of reason why we insist on daily combined measurement of platelet count and P-SEP concentration from the time of ICU admission as follows. First, there is a significant difference between the values of these two markers at ICU admission. And when values remain within the normal range or below the cutoff value, in-hospital survival can be expected in this patient population. However, if values are far outside the normal range or above the cutoff value, the patient population can be considered to have poor prognosis.

Second, when daily combined measurement of these two markers is performed, if their values mostly remain within the normal range or below the cutoff value for approximately 1 week after ICU admission, in-hospital survival can be expected in this patient population.

However, if the values of these two markers remain far outside the normal range or above the cutoff value and do not return to within the normal range or below the cutoff value, in-hospital survival in this patient population is considered unlikely.

In other words, by measuring these two markers daily for approximately 1 week after ICU admission and continuing to follow the daily changes in these values, we may be able to not only predict the patient’s prognosis but may also be able to determine whether the patient has improved or whether the treatment after ICU admission was appropriate. For these reasons, we believe daily combined measurement of platelet count and P-SEP concentrations from the time of ICU admission is important.

In our study, PCT did not reflect the clinical course of COVID-19. A previous report showed that PCT does not tend to increase in viral and fungal infections [32]. Therefore, SARS-CoV-2 infections, in contrast to bacterial infection, usually induce only a modest and delayed increase in circulating PCT concentrations [33].

Generally, concentrations of D-dimer are correlated with the disease severity and predict the risk of thrombosis, the requirement for ventilator support, and mortality [34]. However, in our study, D-dimer was not a useful predictive marker of in-hospital death. We suspect that the reason for this finding is that when patients with COVID-19 develop a severe condition and need to be admitted to the ICU, D-dimer concentrations remain high, regardless of whether they die in the hospital.

### Limitations

This study has some limitations. First, this was a retrospective study and the measurement of each biomarker for each patient was made at different times from the beginning of COVID-19 infection. Second, this study applies only to “severe” COVID-19 cases. Third, ECMO is unavoidable for life support in the management of severe COVID-19-induced acute respiratory distress syndrome. Accordingly, in this study, 54.7% of patients received ECMO management. These patients were usually administered an anticoagulant, mainly unfractionated heparin, upon ICU admission. Because both ECMO therapy and anticoagulants affect hemostatic alterations, their potential effects on the outcomes of this study cannot be ignored. Fourth, bacterial co-infection in COVID-19 ICU patients could partly explain the observed reduced platelet number and may have affected the results. In this study, seven patients (13.0%) had a bacterial infection (five catheter-related infections and two cases of pneumonia) upon ICU admission. On Day 5 of hospitalization, all of these patients had improved bacterial infections, and only one other patient had new ventilator-associated pneumonia. Therefore, although infrequently, bacterial infection may have affected the outcomes of this study. Fifth, because this study focused on measurable markers in our hospital, few data on other markers were examined. Sixth, this study was a relatively short-term single-center study in Fukuoka, Japan. Therefore, further research is required to confirm our findings.

## Conclusion

Daily combined measurement of platelet count and P-SEP concentration from the time of ICU admission appears to be the most valuable set of biomarker measurements for predicting in-hospital death among severe COVID-19 patients and classifying these patients into more detailed severity and risk groups. This finding may lead to improved treatment strategies for patients with severe COVID-19.

## Data Availability

The datasets generated during and/or analyzed during the current study are not publicly available due to protect the privacy of severe COVID-19 patients as much as possible but are available from the corresponding author on reasonable request.
